# P-324. Comparative Efficacy of Alcohol-Based Hand Rub vs. Hand Wash by an In Vivo Cross-Contamination Test Method

**DOI:** 10.1093/ofid/ofae631.527

**Published:** 2025-01-29

**Authors:** James W Arbogast, Cade Comstock, Christopher M Beausoleil, David A Buckley, Steven A Lyon, James Marsden, Donald W Schaffner

**Affiliations:** JW Arbogast Advanced Science Consulting LLC, Akron, Ohio; Nelson Laboratories Bozeman LLC, Bozeman, Montana; Nelson Laboratories Bozeman LLC, Bozeman, Montana; Diversey, Inc., Fort Mill, South Carolina; Chick-fil-A Corporate Support Center, Atlanta, Georgia; RGF Environmental Group, Inc., Denver, Colorado; Rutgers Cook College, Milltown, New Jersey

## Abstract

**Background:**

Hand hygiene is fundamental in reducing the risk of infections, particularly in healthcare and foodservice settings where cross-contamination can easily occur. Understanding the efficacy of different hand hygiene products is crucial for risk assessments, choosing interventions and establishing effective guidance. There is little evidence directly comparing hand wash to alcohol-based hand rub (ABHR) for germ reduction on hands. This study evaluated the in vivo effectiveness of a typical foaming hand wash and ABHR in reducing bacterial load and transfer to food items.

**Figure:**
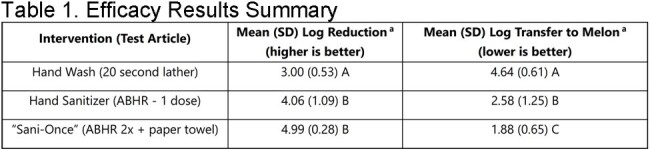
aThe data are expressed as mean results with the standard deviations (SD). Means with different letters in the same column are significantly different (p≤0.05).

**Methods:**

Leading commercially available products (one ABHR and one non-antibacterial foam handwash) were tested with one dose as delivered from their respective automatic wall mounted dispenser using ASTM E2784 (“*Standard Test Method for Evaluation of the Effectiveness of Handwash Formulations Using the Paper Towel (Palmar) Method of Hand Contamination*”). Twelve subjects used all test configurations on the same day in a cross over design. Mean log10 reductions of *Escherichia coli* (ATCC #10536) on hands as well as bacterial counts transferred to melon balls was measured. Analysis of variance was used for statistical data interpretation.

**Figure 1.**
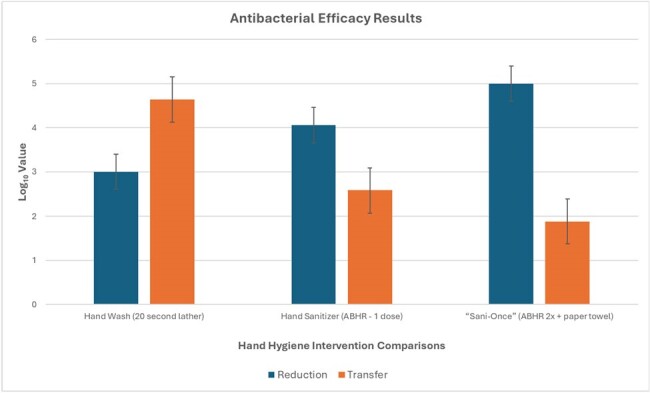
Mean results by hand hygiene intervention type. Error bars indicate 95% confidence intervals.

**Results:**

The results indicate varying degrees of efficacy among the tested hand hygiene products (Table 1). A hand wash with a 20-second lather duration exhibited the smallest mean log reduction (LR) of 3.00 and the highest mean log transfer to melon (4.64). ABHR demonstrated superior efficacy (p≤0.05). One dose of ABHR achieved a mean LR of 4.06 and a mean log transfer to melon of 2.58. "Sani-Once," a combination of two doses of ABHR and paper towel, exhibited the highest efficacy, with a mean LR of 4.99 and a mean log transfer to melon of 1.88 (Figure 1).

**Conclusion:**

This study highlights the importance of choosing appropriate hand hygiene products based on realistic test methods. Disrupting pathogen transmission from hands to food is particularly relevant in both food preparation and service to patients/residents in a healthcare setting. While hand washing can provide substantial bacterial reduction, ABHR intervention (particularly when combined with a paper towel) demonstrates superior efficacy in reducing bacterial load and minimizing transfer to food items.

**Disclosures:**

**James W. Arbogast, PhD**, GOJO Industries Inc.: I was an employee with GOJO until September 1, 2023 (laid off). I have no interaction with them on this abstract and study - so no conflict for me. **David A. Buckley, Ph.D.**, Diversey, a Solenis Company: Advisor/Consultant **James Marsden, Ph.D.**, GOJO: Advisor/Consultant **Donald W. Schaffner, Ph.D.**, GOJO Inc: Advisor/Consultant

